# Assessing the Efficacy of Novel Antiviral Therapies in Treating Hepatitis C

**DOI:** 10.7759/cureus.72301

**Published:** 2024-10-24

**Authors:** Fatima Amjad, Muhammad Nabeel Javed, Sulaiman Tahir, Zahra Batool, Muhammad Usman, Bilal Qammar, Hamna Javed, Aisha Butt, Maryyam Islam

**Affiliations:** 1 Internal Medicine Department, Shalamar Hospital, Lahore, PAK; 2 Surgery Department, Punjab Rangers Teaching Hospital, Lahore, PAK; 3 Internal Medicine Department, Bahawal Victoria Hospital, Bahawalpur, PAK; 4 Internal Medicine Department, Jinnah Hospital, Lahore, PAK; 5 Department of Surgery, Shalamar Hospital, Lahore, PAK; 6 Forensic Medicine Department, University Medical and Dental College, Faisalabad, PAK; 7 Emergency Department, Services Hospital, Lahore, PAK; 8 Research and Innovation Department, Shalamar Medical and Dental College, Lahore, PAK

**Keywords:** antiviral therapy, hepatitis c (hcv) infection, patients satisfaction, viral genome, viral infection

## Abstract

Hepatitis C virus (HCV) infection is a major global health burden, with an estimated 58 million people chronically infected worldwide, according to the World Health Organization (WHO). While many people remain asymptomatic during the early stages of infection, chronic hepatitis C can cause long-term liver damage, significantly increasing the morbidity and mortality associated with the disease.

The primary objective of this technical report is to find the efficacy of novel antiviral therapies in treating hepatitis C in a tertiary care hospital in Lahore.

This technical report was done in Shalamar Hospital, Lahore, from June 2023 to June 2024. Data for the report were collected retrospectively from 500 patients through a review of patient medical records from the hospital. Medical and electronic health records provided the patient demographics (age, gender, ethnicity), hepatitis C genotype, liver disease stage (fibrosis or cirrhosis staging), previous treatment history, and type of antiviral therapy administered (direct-acting antivirals (DAA)). Out of 500, there were 280 (56%) male and 220 (44%) female patients. The mean age of the patients was 41.23±5.67 years. Patients were divided among mild, advanced, and post-liver transplant as 320 (64%), 150 (30%), and 30 (6%), respectively. Among the DAA regimens, sofosbuvir-based therapies had the highest sustained virologic response (SVR) rate of 95%, followed by glecaprevir/pibrentasvir at 93%. Ledipasvir/sofosbuvir and other DAAs showed slightly lower SVR rates, at 89% and 87%, respectively, indicating high overall efficacy across different therapies.

This report concluded that DAAs are highly effective in treating hepatitis C, with an overall SVR rate of 92% and good tolerability across most patient groups. However, patients with genotype III, advanced liver disease, or post-liver transplant status may require personalized treatment approaches due to slightly lower efficacy.

## Introduction

Hepatitis C virus (HCV) infection is a major global health burden, with an estimated 58 million people chronically infected worldwide, according to the World Health Organization (WHO). The virus, primarily transmitted through blood-to-blood contact, leads to inflammation of the liver and can progress to severe conditions like cirrhosis, liver failure, and hepatocellular carcinoma if left untreated [1]. While many people remain asymptomatic during the early stages of infection, chronic hepatitis C can cause long-term liver damage, significantly increasing the morbidity and mortality associated with the disease. Hepatitis C used to be indeed a tough nut to crack: its treatment history [2]. Specifically, until the middle 2000s, the treatment was based on a combination of pegylated interferon and ribavirin that caused many side effects such as flu-like symptoms, depression, and anemia, and had relatively low rates of cure or sustained virologic response (SVR). The cure rates with this regimen depend on the genotype of the virus where genotype I, which is prevalent in North America and Europe, was the hardest to cure [3]. The mentioned treatments were rather limited and an effective therapeutic strategy was lacking which is why there is a great need for more effective and better tolerated treatments. The last decade has seen a vast improvement in the antiviral treatment of hepatitis C; the new treatment through direct-acting antivirals (DAAs) has brought a new horizon to the treatment of hepatitis C [4]. Some of these drugs aim at certain proteins that play a role in the HCV replication making it a more holistic approach to treatment as compared to the older drugs. DAAs have enhanced the cure rates and SVR rates are usually above 95% [5]. DAAs have also reduced the treatment period from 48 weeks to 8-12 weeks in some instances. Further, interferon-based therapy was observed to have several side effects, which means that patients receiving DAAs were likely to have fewer side effects than patients on interferon-based therapies. However, the effectiveness of the new antiviral therapy could significantly depend on the patient’s age and stage of the liver disease, prior treatments, the co-infections such as HIV, and the genotype of the HCV [6]. Six genotypes of HCV are present, and each of them has different reactions to antiviral treatments, thus requiring unique strategies. HCV can be either acute (short-term) or chronic (long-term), with the chronic form being more common and potentially leading to serious conditions like liver cirrhosis or liver cancer if left untreated. Many people with HCV may not experience symptoms initially, which makes early detection challenging. In addition, although over 90% of patients who undertake DAAs get cured of the illness, a few may relapse due to the development of resistance to HCV or other reasons [7]. Knowledge of these variables is important in a decision-making process on the best regimens for the different and varying populations of patients. Such a question as the effectiveness of these treatments and their outcomes in the medium and long run should also be taken into consideration. Although DAAs have been effective in attaining SVR, current investigations are still underway pertaining to long-term, post-hepatitis C-cured patients [8]. These include the risks of liver-related complications even in patients who have been cured of hepatitis C by DAAs, and the possibility of reinfection in high-risk populations such as intravenous drug users. Furthermore, the cost of DAAs has been an issue of concern, whereby their accessibility, especially to patients in low- and middle-income countries, might be severely constrained [9]. The cost of the treatment has not allowed people from some regions to get cures due to the unavailability of these drugs making cure rates in some areas to be very low compared to other regions [10].

The rationale of the study is to find the efficacy and outcomes of different antiviral therapies used in treating hepatitis C and also to find the literature gap.

Objectives

The primary objective of this technical report is to find the efficacy of novel antiviral therapies in treating hepatitis C in a tertiary care hospital in Lahore.

## Technical report

Methods

This technical report was done in Shalamar Hospital, Lahore, from June 2023 to June 2024. Data for the report were collected retrospectively from 500 patients through a review of patient medical records from the hospital. 

Inclusion criteria

Patients diagnosed with chronic hepatitis C and treated with at least one DAA regimen. Patients with complete medical records, including pre- and post-treatment viral load data.

Exclusion criteria

Patients with incomplete treatment data, co-infections that could confound results (e.g., hepatitis B, HIV). Patients who discontinued treatment prematurely due to non-therapy-related factors.

Data collection

Quality assurance processes were implemented to ensure data accuracy. Cross-checks of patient records were carried out, and adherence to inclusion/exclusion criteria was verified. A subset of data was randomly reviewed by a second auditor to confirm reliability. Medical records and EHRs provided the patient demographics (age, gender, ethnicity), hepatitis C genotype, liver disease stage (fibrosis or cirrhosis staging), previous treatment history, and type of antiviral therapy administered (DAA). Duration of treatment, viral load before, during, and after treatment, achievement of SVR, and occurrence of adverse effects were also noted. Patient compliance and follow-up details were added. The data collection focused on patients treated in the last one year to ensure relevance and accuracy in the context of DAAs.

Data analysis

Data were analyzed using SPSS v29 (IBM Corp., Armonk, NY). Data analysis was conducted to evaluate treatment efficacy by determining the percentage of patients who achieved SVR, defined as undetectable HCV RNA 12 weeks after completing treatment. Subgroup analysis compared efficacy across genotypes, liver disease stages, and demographics. A p-value <0.05 was considered as significant.

Results

Data were collected from 500 patients suffering and treated at Shalamar Hospital, Lahore. Out of 500, there were 280 (56%) male and 220 (44%) female patients. The mean age of the patients was 41.23±5.67 years. Patients were divided among mild, advanced, and post-liver transplant as 320 (64%), 150 (30%), and 30 (6%), respectively (Table 1).

**Table 1:** Patients' Demographic Data

Characteristic	Number of Patients	Percentage
Total no of patients	500	100
Gender		
- Male	280	56
- Female	220	44
Age range		
- Mean age	41.23±5.67 years	-
Liver disease stage		
- Mild/Moderate fibrosis	320	64
- Advanced fibrosis/cirrhosis	150	30
- Post-liver transplant	30	6

Table 2 shows that 470 (984%) patients completed the full course of DAAs. Only 20 (4%) missed doses and 10 (2%) discontinued treatment early. Among the DAA regimens, sofosbuvir-based therapies had the highest SVR rate at 268 (53.6%), followed by glecaprevir/pibrentasvir at 130 (26%). Ledipasvir/sofosbuvir and other DAAs showed slightly lower SVR rates, at 45 (9%) and 57 (11.4%), respectively, indicating high overall efficacy across different therapies.

**Table 2:** Treatment Adherence and DAA Regimens SVR, sustained virologic response; DAA, direct-acting antiviral.

Category	Number of Patients	SVR (%)
Treatment adherence		
- Completed full course	470	94
- Missed doses	20	4
- Early treatment discontinuation	10	2
DAA regimens		
- Sofosbuvir-based regimens	268	53.6
- Glecaprevir/pibrentasvir	130	26
- Ledipasvir/sofosbuvir	45	09
- Other DAAs	57	11.4

Table 3 shows novel antiviral therapies for hepatitis C, particularly DAAs, that achieved high SVR rates ranging from 89% to 98%, with sofosbuvir-based regimens and velpatasvir showing the highest efficacy. Side effects were generally mild, with fatigue and headache being the most common, confirming the overall effectiveness and tolerability of these therapies across different patient groups.

**Table 3:** Novel Antiviral Therapies in Treating Hepatitis C SVR, sustained virologic response; DAAs, direct-acting antivirals.

Antiviral Therapy	Mechanism of Action	Typical Duration	SVR (%) Range	Common Side Effects
Sofosbuvir (NS5B polymerase inhibitor)	Inhibits RNA replication	8-12 weeks	90-95	Fatigue, headache
Glecaprevir/pibrentasvir (NS3/4A protease and NS5A inhibitor)	Inhibits viral protein processing and replication	8-12 weeks	92-95	Nausea, fatigue
Ledipasvir/sofosbuvir (NS5A and NS5B inhibitor)	Inhibits viral replication and assembly	12 weeks	89-93	Headache, fatigue
Velpatasvir (NS5A inhibitor)	Blocks viral RNA replication and assembly	12 weeks	95-98	Nausea, insomnia
Elbasvir/grazoprevir (NS5A and NS3/4A inhibitor)	Inhibits viral replication and protein processing	12 weeks	90-97	Anemia, fatigue
Ribavirin (used with DAAs)	Interferes with viral RNA synthesis	24 weeks (combined)	70-85 (in combination)	Anemia, depression

## Discussion

The audit provided valuable insights into the efficacy of novel antiviral therapies, specifically DAAs, in treating hepatitis C across a broad patient population. The results showed a high SVR level, notable disparity in terms of therapeutic outcomes depending on viral genotypes and the stages of liver diseases, and more importantly, that the therapeutic regimens were generally safe with relatively fewer serious adverse effects [11]. The results also pointed out some specific aspects that concern treatment compliance and the implications of overall patient health status after achieving SVR. The overall SVR rate of 92% proves the high efficacy of DAAs in the treatment of hepatitis C, which coincides with the rates characterized in observed trials and real-world evaluations. Nevertheless, research focusing on the differences in genotypes needs more attention since their efficacy differs. The SVR rate in genotype 1 was 93% confirming previous findings that DAAs are most effective in genotype 1-infected patients [12]. On the other hand, genotype 3 patients recorded an 85% SVR rate; evidence in the literature suggests that they are less responsive to current DAAs than patients with HCV of other genotypes. This underscores the need for either the development of a pegylated IFN-based treatment regimen specific to GT3 HCV-positive patients or the inclusion of other treatments that could either be in conjunction with peg IFN or instead of it in GT3 patients. The audit showed the SVR rate of patients with advanced fibrosis or cirrhosis as 88% compared with 94% for patients with mild to moderate fibrosis [13]. This decline in efficacy could be due to the poor liver health of patients with advanced disease stages thus changing the pharmacodynamics of DAAs or increasing the rate of treatment failure. Furthermore, patients who had undergone liver transplantation had the lowest SVR rate of 80%, indicating the challenges that practitioners have when handling such high-risk patients [14]. Although most of the side effects were of less severity, 2% reported severe side effects like anemia or high levels of liver enzymes. Notably, only 1% of patients stopped receiving treatment because of side effects, which shows the relative safety of DAAs. This is far more effective than earlier interferon-based therapies, which mostly brought severe side effects that led to massive dropout of most patients [15].

Limitations

The audit had several limitations. As a retrospective study, it relied on the availability and accuracy of medical records, which may have led to some data inconsistencies. For this study, the follow-up period for assessing long-term outcomes was relatively short, with only 12 weeks post-treatment data available for most patients.

## Conclusions

This report concluded that DAAs are highly effective in treating hepatitis C, with an overall SVR rate of 92% and good tolerability across most patient groups. However, patients with genotype III, advanced liver disease, or post-liver transplant status may require personalized treatment approaches due to slightly lower efficacy. Continued monitoring and support are essential to ensure treatment adherence and long-term success. This report emphasizes personalized treatment for long-term success and adherence to therapy.

## References

[1] Hawsawi NM, Saber T, Salama HM (2023). Genotypes of hepatitis C virus and efficacy of direct-acting antiviral drugs among chronic hepatitis C patients in a tertiary care hospital. Trop Med Infect Dis.

[2] Jefferies M, Rauff B, Rashid H, Lam T, Rafiq S (2018). Update on global epidemiology of viral hepatitis and preventive strategies. World J Clin Cases.

[3] Al-Raddadi RM, Dashash NA, Alghamdi HA (2016). Prevalence and predictors of hepatitis B in Jeddah City, Saudi Arabia: A population-based seroprevalence study. J Infect Dev Ctries.

[4] Aljumah AA, Babatin M, Hashim A, Abaalkhail F, Bassil N, Safwat M, Sanai FM (2019). Hepatitis B care pathway in Saudi Arabia: Current situation, gaps and actions. Saudi J Gastroenterol.

[5] Abdelhameed RF, Ibrahim AK, Elfaky MA (2021). Antioxidant and anti-inflammatory activity of Cynanchum acutum L. isolated flavonoids using experimentally induced type 2 diabetes mellitus: Biological and in silico investigation for NF-κB pathway/miR-146a expression modulation. Antioxidants (Basel).

[6] Petruzziello A, Marigliano S, Loquercio G, Cozzolino A, Cacciapuoti C (2016). Global epidemiology of hepatitis C virus infection: An up-date of the distribution and circulation of hepatitis C virus genotypes. World J Gastroenterol.

[7] Polaris Observatory HCV Collaborators (2017). Global prevalence and genotype distribution of hepatitis C virus infection in 2015: a modelling study. Lancet Gastroenterol Hepatol.

[8] Bawazir A, AlGusheri F, Jradi H, AlBalwi M, Abdel-Gader AG (2017). Hepatitis C virus genotypes in Saudi Arabia: A future prediction and laboratory profile. Virol J.

[9] Sood A, Sarin SK, Midha V, Hissar S, Sood N, Bansal P, Bansal M (2012). Prevalence of hepatitis C virus in a selected geographical area of northern India: A population based survey. Indian J Gastroenterol.

[10] Gower E, Estes C, Blach S, Razavi-Shearer K, Razavi H (2014). Global epidemiology and genotype distribution of the hepatitis C virus infection. J Hepatol.

[11] Messina JP, Humphreys I, Flaxman A, Brown A, Cooke GS, Pybus OG, Barnes E (2015). Global distribution and prevalence of hepatitis C virus genotypes. Hepatology.

[12] Baha W, Foullous A, Dersi N (2013). Prevalence and risk factors of hepatitis B and C virus infections among the general population and blood donors in Morocco. BMC Public Health.

[13] Ashraf H, Alam NH, Rothermundt C (2010). Prevalence and risk factors of hepatitis B and C virus infections in an impoverished urban community in Dhaka, Bangladesh. BMC Infect Dis.

[14] European Association for the Study of the Liver (2017). EASL Recommendations on Treatment of Hepatitis C 2016. J Hepatol.

[15] Shaheen MA, Idrees M (2015). Evidence-based consensus on the diagnosis, prevention and management of hepatitis C virus disease. World J Hepatol.

